# An Evaluation of an Online Brief Mindfulness-Based Intervention in Higher Education: A Pilot Conducted at an Australian University and a British University

**DOI:** 10.3389/fpsyg.2021.752060

**Published:** 2021-10-28

**Authors:** Jennifer Chung, Matthew Edward Mundy, Ian Hunt, Astrid Coxon, Kyle R. Dyer, Stephen McKenzie

**Affiliations:** ^1^School of Psychological Sciences, Monash University, Melbourne, VIC, Australia; ^2^Monash Centre for Professional Development and Monash Online Education, Monash University, Melbourne, VIC, Australia; ^3^Statistical Consulting Service, Monash University, Melbourne, VIC, Australia; ^4^Institute of Psychiatry, Psychology and Neuroscience, King’s College London, London, United Kingdom; ^5^School of Psychological Sciences, The University of Melbourne, Melbourne, VIC, Australia

**Keywords:** mindfulness, student wellbeing, stress, higher education, online intervention, online learning

## Abstract

Mental ill health among higher education students is a well-established problem; therefore, it is imperative to implement preventative approaches to support wellbeing. Blended and fully online education programmes widens access for mature or returning students; however, the psychological wellbeing of this sub-group of students is under-researched. Finally, evaluating wellbeing interventions that meet the needs of university students as well as accessible for online students is required. The aim of this study was to evaluate a brief, online and mindfulness-based intervention to assist the self-management of wellbeing and stress for both online and on-campus higher education students. The total sample included 427 participants (96% psychology students) at Monash University, Australia (*n*=283) and King’s College London (*n*=144), with 152 participants completing the whole study. Participants were allocated to a brief, self-guided, online and mindfulness-based intervention (over the course of one study period; *n*=297), or to a wait-list control group (*n*=148). Baseline and end of semester questionnaires included the 14-item Warwick-Edinburgh Mental Wellbeing Scale, 10-item Perceived Stress Scale and the 15-item Mindful Attention Awareness Scale. Regression modelling revealed the intervention condition accounted for up to 12% of the variability in change in student wellbeing, stress and mindfulness between the start and end of semester (when controlling for baseline). These findings support the implementation of a brief, online and asynchronous mindfulness-based intervention for supporting student mental health and psychological wellbeing. An on-going challenge in practice includes engaging and maintaining student engagement in wellbeing initiatives.

## Introduction

Reduced mental health and wellbeing in university students compared with the general population have been widely reported and is a growing and alarming problem (Stallman, 2010; Storrie et al., 2010; Larcombe et al., 2016; Schofield et al., 2016). In the United States, anxiety in college students has almost doubled in 15years, and over 20% report anxiety negatively impacts their studies (American College of Health Association, 2015). Similarly, in the United Kingdom, approximately 40% of students report symptoms of anxiety and stress (El Ansari et al., 2014; Thorley, 2017; Neves and Hillman, 2019). Likewise, in Australia, it is estimated almost half of university students report distress, and approximately one in four experience severe psychological distress while at university (Stallman, 2010; Larcombe et al., 2016; Rickwood et al., 2016).

While high psychological distress and lower wellbeing in university students are a concern in itself, findings also consistently demonstrate associations with a number of negative academic outcomes. These include decreased academic performance, lower academic self-efficacy, decreased motivation, less ambitious academic plans and lower engagement (Stallman, 2010; Lipson and Eisenberg, 2018). Other outcomes include lower graduation rates and increased likelihood of academic drop-out resulting in financial and economic impacts for institutions (Salzer, 2012; Lipson and Eisenberg, 2018; Papadatou-Pastou et al., 2019). Given the wide range of academic and psychosocial implications for university students and institutions, there is an overwhelming need to address and promote positive student wellbeing and provide preventative resources.

In comparison to the general university student population, evidence suggests that students with certain characteristics experience particularly high levels of distress. This includes students who are female (Stallman, 2010; Schofield et al., 2016), caring for family members (Larcombe et al., 2016) and younger (Stallman, 2010; Schofield et al., 2016). There are mixed findings as to whether students in earlier years of their university study, such as first year (Stallman, 2010), or later years have increased risk of psychological distress (Schofield et al., 2016). The mental health of a sub-group of university students that has been less explored is online learners.

Higher education institutions are increasingly offering blended and online study options and this is seen worldwide. As a consequence of the COVID-19 pandemic, this already expanding study mode is now seeing an even greater accelerated growth. A key benefit for students in online learning is increased choice and more independent time management. This allows them to maintain responsibilities (e.g. caring for family or full-time employment) or lifestyle choices (e.g. living remotely or rurally) that otherwise may need to be compromised if on-campus learning was the only option (Bailey et al., 2015; Johnson, 2015). This flexibility in online learning therefore often attracts mature age students who are returning to study after a prolonged break, after or during workplace employment.

A potential challenge that may be a source of anxiety for ‘returning to study’ adult learners is a lack of technical skills for online study (Roddy et al., 2017; Minutillo et al., 2020) and familiarity with Learning Management Systems (LMS; Saade and Kira, 2009). Other potential challenges include perceived isolation and difficulties balancing work, study and family commitments (Roddy et al., 2017).

Currently, in the world of remote learning, not all things are equal. Students without face-to-face learning often have reduced access to services or experiences that complement their success and wellbeing while in higher education (Chung and McKenzie, 2020). Existing wellbeing initiatives are generally designed for campus attending students, and although there are increasingly more online accessible resources, it is not yet the norm (Roddy et al., 2017; Minutillo et al., 2020). In some cases, online students are not given an equal opportunity to seek support for mental health difficulties from their institution (Chung and McKenzie, 2020), resulting in potentially lower engagement in wellbeing-related initiatives (Minutillo et al., 2020).

Given what we know about student mental health, the potentially decreased access to mental health initiatives targeted for online students, it is essential we create equally accessible opportunities for both on-campus and online students. Universities need to address this gap by providing innovative, mental health preventative measures that utilise a digital platform, to support student wellbeing. A promising wellbeing initiative is mindfulness training.

Mindfulness has gained popularity over the last two decades, is becoming a term that is commonly known by the general public, and research publication on mindfulness has exponentially increased since the beginning of the 21st Century (Creswell, 2017). Mindfulness is the practice of paying attention, being able to direct the attention and accepting whatever we are paying attention to (Kabat-Zinn, 1994, 2003). To be mindful is to be fully connected with present moment reality, and mindfulness techniques, such as breath awareness and the body scan, consist of the practicing of non-judgemental awareness of the present moment reality of the experience of bodily sensations, without reacting to them. It is derived from ancient Buddhist and yoga practices and encourages the deliberate intention of focussing on ones, internal experiences, in the present moment, in a non-judgemental way (Kabat-Zinn, 1994, 2003; Baer, 2003).

Mindfulness-based interventions (MBI) including clinical interventions are becoming increasingly supported by research evidence for their effectiveness in providing a wide range of benefits including improved wellbeing, psychological symptoms, including of stress and anxiety, and improved behavioural regulation (Keng et al., 2011). MBIs have demonstrated a range of positive outcomes in university students including in stress reduction, anxiety and depressive symptoms, mental distress, wellbeing, life satisfaction, relationships and health-related behaviours (Cavanagh et al., 2013; De Vibe et al., 2013; Regehr et al., 2013; Bamber and Kraenzle Schneider, 2016; Dvořáková et al., 2017; Bamber and Morpeth, 2018; Chi et al., 2018; Ma et al., 2019). These findings have been demonstrated in robust randomised controlled trials and supported by meta-analyses (De Vibe et al., 2013; Regehr et al., 2013; Bamber and Kraenzle Schneider, 2016; Dvořáková et al., 2017; Bamber and Morpeth, 2018; Chi et al., 2018; Ma et al., 2019). The evaluation of online MBIs is in its infancy; however, recent meta-analyses found encouraging results for stress reduction (Spijkerman et al., 2016), as well as MBIs that are self-guided (Cavanagh et al., 2014; Ma et al., 2018) and brief, such as three (Shortland et al., 2021) and five minute (Howarth et al., 2019) interventions administered once.

Overall, there is strong support for the use of MBIs given the wide range of potential benefits (for an in-depth review, see Creswell, 2017) and that it is considered relatively non-invasive. Finally, the integration of MBIs into fully online teaching programmes in higher education is yet to be properly explored (Roddy et al., 2017).

The evidence supporting MBIs to achieve a range of positive psychological and health outcomes demonstrates that mindfulness training is a promising intervention to utilise for the prevention of student mental ill health, and promotion of positive mental wellbeing (Světlák et al., 2021). Additionally, the ease of transferring it to an online environment with low cost, relatively minimal time and potentially high reach – makes a MBI an ideal online wellbeing resource allowing universities to provide more inclusive, integrated and accessible resources – including for on-campus and fully online students.

Our primary research question was to evaluate whether there is an effect of a brief asynchronous online MBI on students’ levels of wellbeing, stress and mindfulness across one teaching period. Given time constraints students often express, we were interested in exploring whether a self-guided, 6 and 12week short intervention, could result in benefits that could be achieved with minimal time and effort requirements, and be administered to a large cohort of university students.

As mentioned earlier, there is very limited research on the sub-group of fully online students, and there is research indicating females and younger students may experience greater levels of psychological distress. As such, a secondary research question was to understand whether the impact of a brief MBI would differ for different types of students, specifically in relation to characteristics, such as gender, age, study mode (online or on-campus) and prior experience with mindfulness or meditation.

Therefore, the aim of this study was to evaluate a recently developed online brief MBI on the impact of wellbeing, stress and mindfulness for both on-campus and online students. We aimed to understand the impact of the MBI when provided to students within their LMS, evaluate the ecological validity of an intervention when embedded into their educational context, and evaluate the natural uptake. We conducted this research study utilising two research conditions including a wait-list control and intervention condition.

## Materials and Methods

### Background and Context of the Brief MBI

The resources that have been examined in this pilot study were originally created for and included in an online fourth year psychology course, the Graduate Diploma of Psychology Advanced (GDPA) at Monash University. The resources were written, compiled and led by the Co-Convenor of the GDPA, a male Australian PhD-level psychological researcher (McKenzie, S.) with over three decades of experience engaging in and teaching mindfulness-based practices to university and general populations. The mindfulness exercises were provided to students as an optional wellbeing resource. The framework used, nature of the exercises and length of exercises were created specifically with the educational context in mind. Although the duration of each exercise is shorter than comparative studies, the goal here was to maximise uptake and reduce the perceived burden on students, and thus decisions made when creating the resources was primarily meeting this aim.

### Design

This study adopted a quasi-experimental, pre-test – post-test design. Participants were allocated to the wait-list control condition or the mindfulness intervention condition. Blind allocation was not used, as both researchers and participants were aware of the study condition they had been allocated to. This study was undertaken at two international institutions – Monash University (MU) Australia and King’s College London (KCL).

One of the primary aims of this research was to include participants who were studying fully online programmes. Therefore, programmes and individual subject units that were offered in both an on-campus mode and fully online mode in both MU and KCL were selected. The subject units that were selected and from which students were eligible, include undergraduate and postgraduate students enrolled in psychology, business studies, information technology, public health, nursing and war studies.

For each subject unit and year level (e.g. psychology, first year), one of the study modes (e.g. online) was allocated to the control condition, and the other study mode (e.g. on-campus) was allocated to the intervention condition. When allocating subject units to the study conditions, the research team ensured that an approximately equal number of students were enrolled in each of the study modes and subject units, and that in the overall participant population, an approximately equal number of potential participants were allocated to the control and intervention conditions. No students participated in both study conditions.

The brief MBI evaluated in this study was 12weeks in duration for participants studying in an on-campus mode, and 6weeks in duration for participants studying in a fully online mode. The rationale for this difference in intervention length is that the standard study period (e.g. semester, module and teaching period) is 12weeks for on-campus subjects, and 6weeks for online subjects. This research study evaluated a brief MBI within one study period; therefore, the intervention duration matched the length of the pre-existing study period.

Finally, no minimum intervention completion was required as this brief MBI was examined in an educational context. We aimed to create a naturalistic setting where students would not be required to participate in wellbeing activities, and instead, completion of sessions would be entirely voluntary. Asking participants to complete a minimum number of sessions, or excluding those who did not participate would not evaluate the ecological validity of this MBI.

### Participants

Participants were recruited from MU and KCL between January and June 2019. Participants who were enrolled as an on-campus student attended a city campus in Melbourne, Australia or London, England. Participants who were enrolled as an online or distance learning student completed their studies entirely online and there is no requirement for them to attend a university campus during their enrolment.

Participation was voluntary and the only inclusion criteria was that participants were required to be over the age of 18. Participants were given the opportunity to enter a draw to win a $50 AUD/£20 gift voucher.

**Figure 1:**
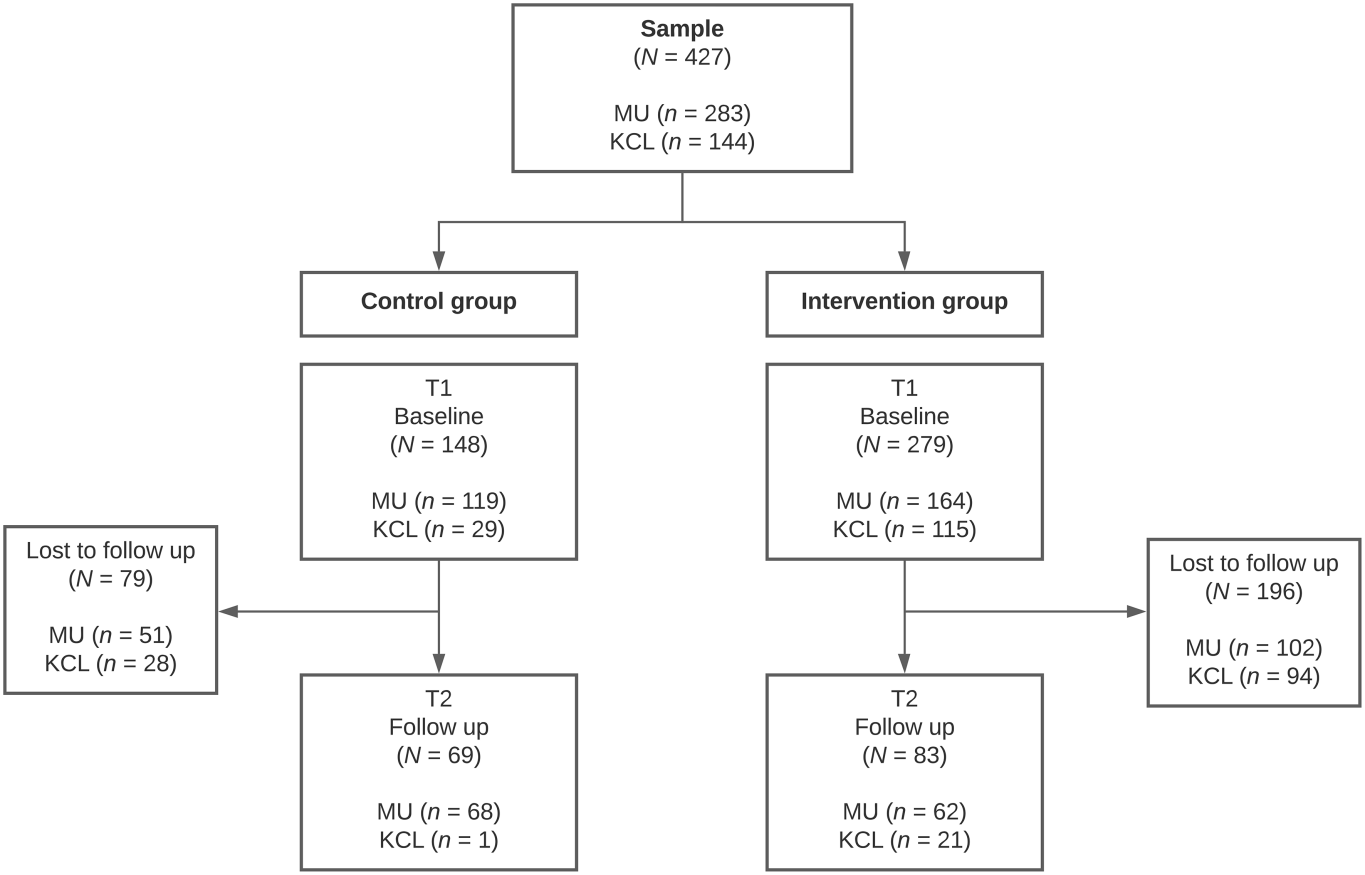
Participant flow chart representing the sample size of each condition and participants lost to follow-up. MU, Monash University Australia; KCL, King’s College London; T1, Time 1; and T2, Time 2.

The flow of participants through the study is illustrated in Figure 1. A total of 427 participants (283 MU, 144 KCL) provided consent to participate in the study and completed the baseline survey. During the study period, 153 (54.1%) and 122 (84.7%) participants at MU and KCL, respectively, did not complete the entirety of the study.^1^ Full datasets were obtained for 152 (total 35.6%, MU *n*=130, KCL, *n*=22) participants. Full datasets were obtained for 69 participants in the control group, and 83 participants in the intervention group. Full random allocation and stratification were not possible, yet baseline comparisons revealed participants were well matched (see Table 1). Majority of the sample (96%) was psychology students. The demographics and characteristics of the sample are shown in Table 1.

**Table 1:** Participant characteristics as a percentage of the sample.

	Total	Control	Intervention
	(*N* =427)	(*n* =148)	(*n* =279)
**Institution**			
MU	283 (66%)	119 (80%)	164 (59%)
KCL	144 (34%)	29 (20%)	115 (41%)
**Age**			
18–25	198 (47%)	91 (62%)	107 (39%)
26–35	97 (23%)	21 (14%)	76 (27%)
36–45	77 (18%)	21 (14%)	56 (20%)
46+	53 (12%)	14 (10%)	39 (14%)
**Gender**			
Female	351 (82%)	118 (79%)	233 (84%)
Male	70 (16%)	29 (20%)	41 (15%)
Non-binary	2 (0.5%)	1 (1%)	1 (0.5%)
**Study mode**			
Online	254 (60%)	62 (42%)	192 (67%)
On-campus	173 (40%)	86 (58%)	87 (32%)
**Course level**			
Undergraduate	283 (66%)	119 (80%)	164 (59%)
Postgraduate	144 (34%)	29 (20%)	115 (41%)
**Discipline**			
Psychology	406 (96%)	144 (98%)	262 (94%)
Information Technology	9 (2%)	3 (2%)	6 (2%)
Public health	5 (1%)		5 (2%)
Nursing	5 (1%)		5 (2%)
**Prior practice**			
Meditation (Yes)	267 (73%)	62 (72%)	205 (74%)
Mindfulness (Yes)	176 (71%)	59 (69%)	117 (72%)

*MU, Monash University Australia; KCL, King’s College London. Percentages are based on valid percent*.

### Materials

#### Original Development of the Mindfulness Exercises

##### Creation of the Exercises

As provided in the Section ‘Background and Context of the Brief MBI’, the current examination of a pilot of a brief MBI was based on the resources provided to students in an existing course, the GDPA at MU. The context in which these resources were created must be considered when evaluating this pilot as core decisions in this study was made with the educational context in which we aim to implement this MBI in the future.

##### Compilation of Exercises

The intervention resources for MU participants were written, compiled and led by a male Australian PhD-level psychological researcher (McKenzie, S.) with over three decades of experience engaging in and teaching mindfulness-based practices to university and general populations. During the design of the research study, key stakeholders at the research institute at KCL deemed that it was necessary to replicate the intervention materials specifically for their cultural context being a UK University. Therefore, the mindfulness exercises were re-recorded by Coxon, A., a female, British PhD-level psychologist, teaching fellow in the school and research institute, and research investigator in this project. More specifically, Coxon, A., holds a Master of Science in Health Psychology, completed training in mindfulness and Acceptance & Commitment Therapy, and university modules in therapeutic interventions including Cognitive Behaviour Therapy and mindfulness.

#### The Brief Mindfulness-Based Intervention

##### Mindfulness Framework and Content

The brief MBI was 6weeks in duration for participants studying in an online mode, and 12weeks for participants studying in an on-campus mode. The entirety of the intervention programme was asynchronous and did not include any face-to-face components. A brief description of mindfulness was provided to participants in the explanatory statement prior to commencing the research study.

To access the intervention materials, participants were provided access to a dedicated LMS site that contained only the intervention materials. Participants logged into their university’s LMS and then navigated to this dedicated LMS site. Within the brief MBI LMS site, no introduction to mindfulness was provided. The contact details of the researchers were provided on the site homepage. In equal intervals across the semester (weekly for 6-week semesters, and fortnightly for 12-week semesters), each of the six mindfulness exercises were released and became available. The exercises were released at approximately equal intervals across the week (e.g. every Monday). Each time the exercises were released and became available, announcements were sent to participants *via* the LMS site (resulting in an email notification).

The mindfulness sessions consisted of a total of six, short (1–2min), pre-recorded audio-guided mindfulness practices, each accompanied by soft instrumental background music. The mindfulness sessions were as follows: (1) feel the body 1 (focussing on the present moment and awareness of internal experiences, version 1), (2) feel the body 2 (focussing on the present moment and awareness of internal experiences, version 2), (3) sounds (focussing awareness on sounds), (4) sights (focussing awareness of sights), (5) the breath (focussing awareness on breathing) and (6) connection (focussing on the connection and awareness of current experiences).

As the mindfulness exercises were accessible online, *via* the dedicated LMS site, participants needed Internet access to first access the audio files. Hereafter, participants had the option of navigating to the LMS site each time to listen to the sessions, or they could download the audio file directly to their device. Transcripts of the audio files were also available for download. Participants were not restricted to listening to the audio files in any particular location, such as the university campus.

Participants in the online study mode (6-week semesters) and on-campus study mode (12-week semesters) were provided the same mindfulness materials, and only the time between each of the exercises was released, differed. In the written instructions to participants, they were encouraged to participate in the exercises as often as they liked, could repeat the exercises multiple times, and reminders *via* the LMS site were also sent throughout the study. Participants were not asked to record the number or amount of times they completed the exercises.

It must be noted that an introduction to mindfulness nor the exercises was not provided to participants beyond the explanation provided in the explanatory statement at the commencement of the research study. This decision was due to the nature of this study examining the efficacy of a brief introduction to mindfulness, as well as the intention to evaluate the efficacy of existing mindfulness resources, such as in the GDPA.

### Measures

#### Questionnaire

Participants created a unique identifier allowing researchers to link survey responses from time 1 (T1) and time 2 (T2). The baseline (T1) questionnaire consisted of basic demographic information including age, gender, study mode and course level. Participants were asked (yes/no) if they had meditated or completed mindfulness before. Participants then completed the following three validated scales. The post-intervention questionnaire (T2) included the same three scales. At MU, engagement with the mindfulness intervention was measured by a single self-report item in the follow-up survey. The item asked ‘how many of the six mindfulness exercises did you complete?’, choices ranged from 0 to 6. This item was not included in the survey at KCL.

##### Warwick-Edinburgh Mental Wellbeing Scale

The Warwick-Edinburgh Mental Wellbeing Scale (WEMWBS) is a 14-item scale measuring wellbeing over the past 2weeks (Tennant et al., 2007). Items are measured on a 5-point Likert scale from *1=none of the time* to *5=all of the time*. Due to a procedural error, participants in the MU sample completed 12 items in the WEMWBS (excluding items 12 & 13). To appropriately combine and compare MU and KCL datasets, items were summed and averaged (rather than using a summed score). Higher average WEMWBS scores indicate increased wellbeing. Cronbach’s alpha has been reported as 0.89–0.91 in university student and population samples, as well as high test-retest reliability (*α*=0.82; Tennant et al., 2007). Cronbach’s alpha for MU sample based on 12 items was 0.89, and at KCL with 14 items was 0.90. Both samples demonstrated high internal consistency.

##### Perceived Stress Scale

The Perceived Stress Scale (PSS) is a commonly used 10-item scale measuring the perception of stress (Cohen et al., 1983). Participants rated items on a 5-point Likert scale (0=never to 4=very often), over the past month. Four items are reverse scored, all items are summed to produce a total PSS score between 0 and 40. PSS scores ranging from 0 to 13, 14 to 26 and 27 to 40 indicate low, moderate and high perceived stress, respectively. Cronbach’s alpha indicates high reliability with alpha coefficients of 0.84–0.86, and test-retest reliability correlation of 0.85 in college students (Cohen et al., 1983). Similarly, Cronbach’s alpha coefficient in this sample was 0.89 demonstrating high reliability.

##### Mindful Attention Awareness Scale

The Mindful Attention Awareness Scale (MAAS) is a 15-item scale measuring dispositional mindfulness (Brown and Ryan, 2003). Items are rated on a 6-point Likert scale from *1=almost always* to *6=almost never*. Items are summed and averaged, with higher scores reflecting higher dispositional mindfulness. In the current sample, Cronbach’s alpha for MAAS was 0.89, demonstrating high internal consistency, similarly to Brown and Ryan (2003; *α*=0.92).

### Procedure

Ethical approval was obtained from the human research ethics committees from both MU and KCL prior to the start of the study. Participation in this study included the collection of additional data unrelated to the hypotheses here; thus, only data that are analysed in this study are currently presented. At the commencement of the semester, course convenors and administrators invited students to voluntarily participate *via* the programme’s LMS site. The research team provided the invitation to ensure consistency.

Potential participants were invited to self-enrol in the LMS site created for this study. The explanatory statement was provided which detailed the nature of the research study evaluating a brief, online mindfulness intervention. The explanatory statement indicated to students which experimental condition they would receive (wait-list control or intervention), were provided a brief and general definition of mindfulness, were informed of the type of mindfulness activities, the duration of the intervention and study, and finally the types of questions asked in the survey. Participants then provided consent and completed the baseline (T1) survey *via* an online survey platform. Participants were not able to be identified from their response, as responses were anonymous.

Participants in the control condition were not required to complete any further tasks until the end of the study period. Participants in the intervention condition were given access to the MBI LMS site. Participants were encouraged to complete the brief MBI for the course of their semester (12weeks for on-campus participants; 6weeks for online participants); however, here was no minimum required participation in the mindfulness intervention. At the end of the study (and semester), all participants were invited to complete the post-intervention (T2) survey. Participants in the wait-list control condition received access to the mindfulness intervention following the conclusion of the research study.

## Statistical Procedure

To determine if drop-out of the study was associated with participants’ baseline levels of the outcome measures, three independent measures *t*-tests were conducted between a group of participants that completed only the baseline survey, and a group that completed both baseline and post-intervention surveys. Baseline levels of wellbeing, stress and mindfulness in the control and intervention group were compared using three independent measures *t*-tests.

The main outcome variable of interest was the difference between scores at follow-up compared to baseline (i.e. T2 scores − T1 scores), computed for each outcome variable, which we refer to as ‘change’. Change was examined in regard to the size (i.e. the size of the units between the two-time points) and direction (i.e. changes that were positive or negative in outcome value). Change values that were positive indicated increased scores, and negative change values indicated decreased scores on the outcome measure.

Independent *t*-tests were used to compare group differences on all binary predictor variables on levels of change in wellbeing, stress and mindfulness between T1 and T2. Hedge’s *g* was used to calculate effect size and is recommended when sample sizes are small and unequal. For the main analysis and to explore the impact of the intervention on participants levels of wellbeing, stress and mindfulness, a series of regression models were applied. The models predicted the variation of change in outcome between T1 and T2 accounted for by the intervention and participant demographics and characteristics. Baseline levels of the outcome variable at T1 were used as a covariate in the models. Our primary focus is on reporting the variance accounted for by the condition; however, we also present the full model that explains the variance accounted for by various variables.

## Results

### Descriptive Statistics and Baseline Comparisons

#### Drop-Out Analysis

The independent samples *t*-tests conducted between participants who completed the study and those that withdrew (i.e. did not complete the follow-up survey) yield no evidence that participants differed significantly on any of outcome measures of WEMWBS, PSS and MAAS (see Supplementary Material 1).

#### Baseline Comparisons

Three independent samples *t*-tests (with a Bonferroni adjustment of *p*=0.017) were conducted to compare baseline scores between the control and intervention groups (Table 2). Participants in the control and intervention groups did not differ on WEMWBS, PSS and MAAS scores at baseline, *t*(422)=0.94, *p*=0.35, 95% CI (−0.06, 0.17), Hedge’s *g*=0.10; *t*(414)=0.47, *p*=0.64, 95% CI (−1.02, 1.65), Hedge’s *g*=0.05; *t*(418)=0.46, *p*=0.64, 95% CI (−0.12, 0.19), Hedge’s *g*=0.05, respectively. Secondary baseline comparisons included independent samples *t*-tests between groups of participant demographics (see Supplementary Material 2).

**Table 2:** Outcome measures at baseline and post-intervention for the control and intervention group.

	Control		Intervention	
	Baseline	Follow-up	Baseline	Post-intervention
	(*n* =144)	(*n* =68)	(*n* =272)	(*n* =79)
WEMWBS	3.36 (0.58)	3.00 (0.65)	3.32 (0.56)	3.32 (0.60)
PSS	20.35 (6.70)	22.26 (7.10)	20.04 (6.51)	19.10 (6.85)
MAAS	3.70 (0.78)	3.46 (0.92)	3.67 (0.74)	3.89 (0.83)

*Data are Mean (SD). WEMWBS, Warwick-Edinburgh Wellbeing Scale; PSS, Perceived Stress Scale; and MAAS, Mindful Attention Awareness Scale*.

Although participant characteristics in the sample were mostly balanced, there was an uneven distribution of participant ages between the online and on-campus study modes. Of the on-campus participants, almost all (99%, *n*=171) were younger people (i.e. 18–35), whereas approximately half of the online participants (*n*=129) were aged 36 and over. Further evidence of confounding between the age and study mode was evidenced by significant differences in terms of baseline outcome measures (see Supplementary Material 2). As a result, we analyse regression models that include these variables separately.

### Evaluation of the Mindfulness-Based Intervention

Fifty-eight respondents completed the follow-up self-report survey item measuring the number of mindfulness exercises completed. Intervention engagement as measured by the number of mindfulness exercises completed ranged from none to six (*M*=4.34, *SD*=2.02). Fifty percent of respondents completed all six mindfulness practice, and 7% did not complete any.

**Figure 2:**
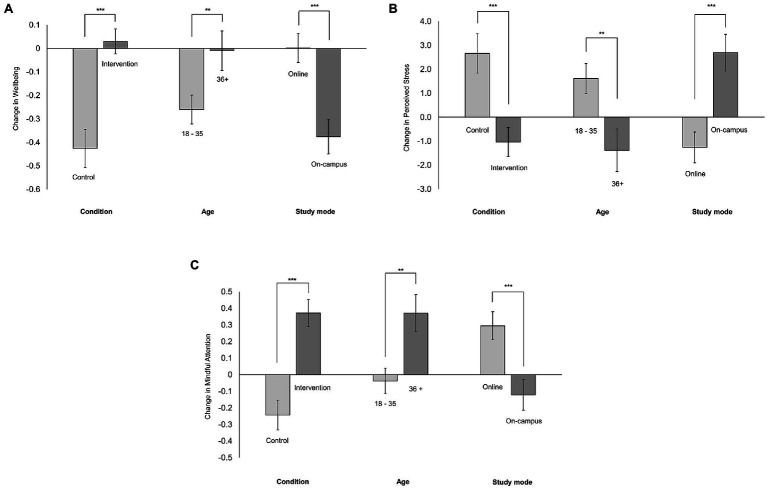
Group differences for condition, age and study mode in **(A)** wellbeing, **(B)** stress and **(C)** mindful attention. Change=Time 2−Time 1. Positive change values indicate an increase in scores from Time 1 to Time 2. Negative change values indicate a decrease in scores from Time 1 to Time 2. Standard errors are represented in the figure by the error bars attached to each column. ^**^p<0.01 and ^***^p<0.001.

Group differences by participant characteristics and demographics on change in WEMWBS, PSS and MAAS scores, between T1 and T2, were explored (see Supplementary Material 3). Significant group differences were found in condition, age and study mode for each of the three outcome measures. Non-significant group differences were found for the remaining variables (institution, gender, level, meditation or mindfulness experience). The size and direction of mean change in outcome scores for significant group differences (condition, age and study mode) are depicted in Figure 2.

Intercorrelations between the three continuous outcome measures, WEMWBS, PSS and MAAS at baseline, and the change between T1 and T2 are presented in Table 3. The correlations between baseline scores and change in scores for each outcome are negative and moderate (Table 3). Lower baseline scores were associated with positive change values between T1 and T2. Conversely, higher baseline scores were associated with negative change values between T1 and T2. Extreme baseline scores were more likely to show a bigger change than participants closer to the mean range of the scale.

**Table 3:** Intercorrelations between outcome measures.

No.	Measure	1	2	3	4	5	6
1.	Baseline WEMWBS	1					
2.	Change in WEMWBS	**−0.43** ^***^	1				
3.	Baseline PSS	**−0.74** ^***^	**0.23** ^**^	1			
4.	Change in PSS	**0.37** ^***^	**−0.76** ^***^	**−0.41** ^***^	1		
5.	Baseline MAAS	**0.54** ^***^	**−0.40** ^***^	**−0.52** ^***^	**0.37** ^***^	1	
6.	Change in MAAS	−0.14	**0.64** ^***^	−0.002	**−0.61** ^***^	**−0.37** ^***^	1

*WEMWBS, Warwick-Edinburgh Wellbeing Scale; PSS, Perceived Stress Scale; and MAAS, Mindful Attention Awareness Scale. Change=Time 2−Time 1. Significant findings are in bold*.

** *p<0.01 and*

*** *p<0.001*.

#### Regression Predicting Change in Outcome

To evaluate the intervention, and the relationship between participant demographics and characteristics on outcomes of wellbeing, stress and mindfulness, multivariate regression modelling was applied. Based on the significant one-to-one differences depicted in Figure 2 (as seen in Supplementary Material 3), straightforward linear regression models were created by adding the following variables in order: baseline scores corresponding to the outcome variable, condition and *either* age or study mode. Regression coefficients (with confidence intervals and values of *p*), *R*^2^, adjusted *R*^2^, change in *R*^2^ and significance values for the models for each outcome measure are reported in Table 4.

**Table 4:** Models predicting change in wellbeing, stress and mindful attention.

**Model for predicting change in WEMWBS**												
	Model 1						Model 2					
*N*=151	*B* (95% CI)	Value of *p* for *B*	*R* ^2^	Adj. *R*^2^	Δ*R*^2^	*F*	*B* (95% CI)	Value of *p* for *B*	*R* ^2^	Adj. *R*^2^	Δ*R*^2^	*F*
Constant	1.10 (0.59, 1.61)	<0.001					1.42 (0.88, 1.96)	<0.001				
Baseline WEMWBS	−0.46 (−0.61, −0.32)	<0.001	0.18	0.18		**33.27** ^***^	−0.47 (−0.61, −0.33)	<0.001	0.18	0.18		**33.27** ^***^
Condition	0.35 (0.18, 0.52)	<0.001	0.29	0.28	**0.10** ^***^	**30.05** ^***^	0.27 (0.09, 0.45)	0.004	0.29	0.28	**0.11** ^***^	**30.05** ^***^
Age	0.27 (0.09, 0.46)	0.004	0.33	0.31	**0.04** ^**^	**23.90** ^***^	–	–	–	–	–	–
Study mode	–	–	–	–	–	–	−0.34 (−0.52, −0.16)	<0.001	0.35	0.34	**0.06** ^***^	**26.54** ^***^
**Model for predicting change in PSS**												
	**Model 1**											
*B* (95% CI)	Value of *p* for *B*	*R* ^2^	Adj. *R*^2^	Δ*R*^2^	*F*						
Constant	12.26 (9.29, 15.23)	<0.001										
Baseline PSS	−0.44 (−0.57, −0.31)	<0.001	0.17	0.16		**29.92** ^***^						
Condition	−2.80 (−4.56, −1.05)	0.002	0.24	0.24	**0.08** ^***^	**23.90** ^***^						
Age	−4.02 (−5.96, −2.08)	<0.001	0.33	0.31	**0.08** ^***^	**23.26** ^***^						
**Model for predicting change in MAAS**												
	**Model 1**						**Model 2**					
*B* (95% CI)	Value of *p* for *B*	*R* ^2^	Adj. *R*^2^	Δ*R*^2^	*F*	*B* (95% CI)	Value of *p* for *B*	*R* ^2^	Adj. *R*^2^	Δ*R*^2^	*F*
Constant	1.07 (0.55, 1.60)	<0.001					1.20 (0.62, 1.78)	<0.001				
Baseline MAAS	−0.38 (−0.52, −0.24)	<0.001	0.13	0.13		**22.61** ^***^	−0.34 (−0.48, −0.20)	<0.001	0.13	0.13		**22.61** ^***^
Condition	0.47 (0.25, 0.69)	<0.001	0.26	0.25	**0.12** ^***^	**25.20** ^***^	0.45 (0.21, 0.69)	<0.001	0.26	0.25	**0.12** ^***^	**25.20** ^***^
Age	0.45 (0.21, 0.69)	<0.001	0.32	0.31	**0.06** ^***^	**22.85** ^***^	–	–	–	–	–	–
Study mode	–	–	–	–	–		−0.27 (−0.51, −0.03)	0.028	0.28	0.27	**0.02** ^*^	**18.89** ^***^

*Each variable was entered into the model in the order as presented. The B column (and associated values of p) has values from the final model with all variables included. The remaining columns are marginal results that gauge the effect of including each variable in sequence (for example, the ΔR^2^ for condition is the improvement of overall model fit from adding the condition variable to a model that only uses the baseline variable). WEMWBS, Warwick-Edinburgh Wellbeing Scale; PSS, Perceived Stress Scale; and MAAS, Mindful Attention Awareness Scale. Change=Time 2−Time 1. CI, confidence interval. Adj, adjusted. Condition: 0=control, 1=intervention. Age: 0=18–35, 1=36+. Study mode: 0=online, 1=on-campus*. Bold is used to highlight and emphasise key data.

* *p<0.05;*

** *p<0.01 and*

*** *p<0.001*.

#### The Effect of the Intervention on Wellbeing

Baseline WEMWBS scores as a covariate accounted for 18% of the variance in change in WEMWBS scores between T1 and T2. Following this, an additional 11% of variance was accounted for by condition, with a small to medium effect size (*f*^2^=0.13). Finally, when study mode was added, an additional 6% was accounted for. In combination, the three predictor variables explained 35% of the variance in change in WEMWBS scores between T1 and T2. According to Cohen (1988), a combined effect of this magnitude can be considered large (*f*^2^=0.54). In Model 1 and 2, respectively, the regression coefficient of the intervention condition predicts a 0.35 unit or 0.27 unit increase in wellbeing change. This means that after controlling for the other predictor in the model (Baseline WEMWBS), being in the intervention condition will result in a predicted 0.35 unit increase in change in WEMWBS.

#### The Effect of the Intervention on Perceived Stress

Baseline PSS accounted for 17% of the variability in change in PSS scores. After controlling for baseline PSS, the study condition accounted for 8% of the variance, and the effect size was small, *R*^2^=0.08, *f*^2^=0.087. Finally, age accounted for an additional 8% of the variance, with the full model (baseline PSS, study condition and age) accounting for a total of 33% of change in PSS between T1 and T2, reflecting a large effect size (*f*^2^=0.49). When controlling for other variables, the intervention condition will result in a 2.80 unit decrease in prediction of change in PSS.

#### The Effect of the Intervention on Mindfulness

After controlling for baseline MAAS scores which accounted for 13%, the study condition explained 12% and age accounted for 6% of the variability in change in MAAS scores. When examining the effect of the study condition (accounting for baseline MAAS scores), there was a medium effect size (*f*^2^=0.14). Combined, the full model predicted 32% of the variability in change in MAAS scores between T1 and T2, with a large effect size (*f*^2^=0.47). Similar to the other outcome measures, a 0.47 unit increase in mindfulness change is predicted when in the intervention condition and other variables are controlled for.

## Discussion

This quasi-experimental study evaluated the impact of a brief MBI on university students’ levels of wellbeing, stress and mindfulness over the course of one semester. Participants were allocated to one of two conditions: wait-list control and intervention condition. This brief MBI was piloted across undergraduate and postgraduate, on-campus and online students in psychology, IT, public health and nursing at two international institutions in Australia and Britain. As our sample included online students, our sample had a higher proportion of mature age students in comparison with similar studies. Majority of the final sample (96%) was made up of psychology students.

Three validated measures of wellbeing, perceived stress and mindful attention were administered at both baseline and follow-up. Overall, we found the mindfulness intervention significantly improved all three outcome measures compared with the control condition. After controlling for baseline levels, age and study mode, the intervention condition predicted between 8 and 12% variability in change (small–medium effect size) in outcome between the start and end of the semester (Table 4). The other demographics and student characteristics were not significant predictors of outcome change (see Supplementary Material 3). Finally, we acknowledge that compliance and study retention in the overall sample were low, with 46% at MU and 15.3% at KCL, of participants completing the follow-up survey, indicating that it was also a particularly large issue at KCL. Possible reasons for low study retention are discussed in the limitations.

Our results support the effectiveness of the *condition*, a brief and self-managed MBI in students in two universities in two different countries, on positive improvement in wellbeing, stress and mindful attention over the course of the semester.

### The Evaluation of the Intervention

We found participants in the intervention condition demonstrated significantly improved outcomes on all three measures including wellbeing, stress and mindfulness, compared to control participants. After controlling for participants’ baseline levels of the measure, the intervention condition accounted for between 8 and 12% of the variability in change between T1 and T2, with the intervention contributing the most to change in mindfulness (12%) and wellbeing (11%), and the least in perceived stress (8%; Table 4).

As seen in Table 2, group differences showed that on average, participants in the intervention demonstrated positive increases in wellbeing and mindfulness across the semester, whereas levels of wellbeing and mindfulness dropped in participants in the control. These group differences were large. Importantly, participants’ levels of perceived stress in the control condition increased from start to the end of semester, whereas average levels of stress levels decreased in those receiving the intervention and the group difference was of a moderate effect size. The intervention significantly impacted all three outcome measures in this study.

At the start of semester, the sample presented with a ‘moderate’ level of perceived stress, as measured by the PSS (Cohen et al., 1983). While by the end of the semester, control participants’ perceived stress increased, and intervention participants decreased, both groups were still presenting ‘moderate’ levels of stress. That is, control and intervention participant scores on the PSS increased and decreased, respectively; however, neither groups reached what would be considered ‘high’ (i.e. concerning) or ‘low’ stress as indicated by the authors of the measure.

The combination of findings relating to self-reported stress and mindfulness is consistent with a large narrative review of 57 articles (Bamber and Kraenzle Schneider, 2016) where over 75% of the studies that examined stress as an outcome reported reductions after mindfulness related interventions. Similarly, 91% of the studies that examined mindfulness reported increases after the MBI (Bamber and Kraenzle Schneider, 2016). Bamber and Morpeth’s (2018) meta-analysis found MBIs for college students with greater number of mindfulness sessions (eight or more) showed greater reductions in anxiety, interestingly however, session duration and the overall amount of time spent meditating was not significant. Although we were unable to measure the impact of the amount of practice, our findings are consistent with Bamber and Morpeth (2018)’s findings. Whilst the individual mindfulness sessions in our intervention were brief, perhaps the overall number of sessions completed was enough to show intervention effects. Finally, our intervention effects were not dependent on gender. This is in contrast to general college students (Bamber and Kraenzle Schneider, 2016; Rojiani et al., 2017) and, in a population similar to ours of medical and psychology students (De Vibe et al., 2013), where it was reported only women showed improvements or benefits.

These results demonstrate that albeit brief, the MBI conducted in this study helped to maintain levels of wellbeing and mindfulness in students, as well as prevent and decrease perceived stress levels. Overall, our findings are very encouraging and consistent with systematic and meta-analytic reviews examining MBIs in student populations (Bamber and Kraenzle Schneider, 2016; McConville et al., 2017; Bamber and Morpeth, 2018; Halladay et al., 2019).

### Other Predictive Variables

Aside from the condition variable that represents the intervention, we found that three other variables predict changes in student outcomes between T1 and T2: baseline levels of the outcome measures at T1, age and study mode.

Negative correlations (Table 3) and negative regression coefficients (Table 4) revealed participants’ baseline levels of a particular outcome measure were negatively associated with the change in the corresponding outcome between T1 and T2. In other words, a lower score on the wellbeing, stress and mindful attention measures is correlated with change values in a positive direction between T1 and T2, with a converse relationship for higher baseline scores (Table 3). Some of this correlation may be due to ‘regression to the mean’.

Being 36years old or older was positively related to greater increases in change in wellbeing and mindfulness, and greater reduction in perceived stress symptoms (Figure 2). Our findings support Stallman (2010) and Schofield et al. (2016) where younger students were more at risk of developing stress symptoms, and decreased wellbeing by the end of the semester. Suggesting perhaps that older students may be less susceptible to or more equipped to handle study-related stresses.

Lastly, our study is one of the first to our knowledge to compare the psychological outcomes of students studying in on-campus or blended/fully online modes (Minutillo et al., 2020). We found significant differences between the study modes, and interestingly, online students experienced a higher likelihood of reduction in change values, indicating that by the end of the semester, they had higher wellbeing and mindfulness, and decreased stress (compared to baseline) in comparison with their on-campus counterparts (Figure 2). However, this finding is confounded by the intervention duration as online students participated in the 6-weeks version compared to 12weeks for on-campus students. Further disentangling of this finding as well as understanding *why* online students saw greater positive change needs to be understood in future research.

The relationship between the change in outcomes with age and study mode is confounded for two reasons. First, there is significant correlation between age and study mode because nearly all older participants were studying online. Secondly, there is an imbalance of baseline outcome measures between age and study mode groups. Despite these issues, we believe the separate regressions in Table 4 and the clear correlations in Figure 2 identify a significant relationship between the change in outcomes and both age and study mode. This is a significant contribution to the literature on the mental health of online students as little has been published in this area to date.

Conversely, the lack of group differences between students in Australia and Britain provides evidence that the student characteristics and profiles at the two universities (and potentially countries) are comparable, and thus supports the implementation of similar wellbeing-related interventions. Similarly, group differences were not found between remaining variables of prior experience practicing mindfulness or meditation, and level of study (consistent with the lack of consensus; Stallman, 2010; Schofield et al., 2016).

### Interactions Between Covariates

The simplicity of the regression models (Table 4) enables straightforward *interpretations* of how the condition variable is related to the changes in the outcome measures. However, these models are not definitive and the true relationships may be more complex. In Supplementary Material 4, we have tested more complex models. The underlying coefficients in these models cannot be so easily interpreted (including because of multicollinearity between the predictor variables), but the additional results suggest that there are three important features of our data which cannot be identified in Table 4.

Firstly, the best predicative model for the change in any particular outcome measure may involve baseline measures from the other outcome variables. This is evidenced by the significant intercorrelations between the three outcome measures (Table 3). Secondly, there may be significant interaction effects between condition and the baseline outcome variables (in other words, for any individual the change in an outcome due to the intervention may depend on the value of their baseline measures). Thirdly, despite any apparent model complexity, there remains clear evidence that the intervention is related to the *changes* in all three of the outcome variables.

### Strengths, Limitations and Future Research

#### Strengths

As suggested by Bamber and Kraenzle Schneider (2016), evaluations of MBI’s can lack mindfulness as an outcome measure, and thus, our inclusion of MAAS was a strength in the current study. Although in future research, a multi-dimensional measure of mindfulness is preferred. Secondly, our quasi-experimental pre-post study design included a wait-list control comparison. Thus, we were able to compare not only the changes in outcome as a consequence of the intervention, we were also able to compare against a control group to understand the ‘normal’ trajectory of wellbeing, stress and mindfulness levels between the start and end of the semester. Evaluating the effect of the intervention based on change scores rather than T2 scores is an unbiased estimate of the mean differences between groups.

A final but significant strength in our study was the direct recruitment of fully online students. Our total sample was made up of 60% online students and thus meets our goal of providing increased access to wellbeing enhancing resources to non-campus students. This study gives us insights into the wellbeing, stress and mindfulness of a sub-group of students who have been under-researched (Minutillo et al., 2020).

#### Limitations

A major limitation of the study findings is the percentage of participant drop-out in the study, and in particular, the significantly low participant retention at KCL. Some possible reasons we propose for low participant retention include participants that may have experienced negative effects (and the lack of follow-up for participants who experienced this), and the nature of the intervention as it was fully online including a lack of physical connection, community and fully asynchronous nature. It is necessary to explore this in future studies as we currently do not have the research data to validate these possible reasons.

Specifically, in regard to KCL attrition and its stark contrast compared to MU attrition, we suggest that this can largely be explained by contextual factors with mindfulness and wellbeing being a normalised concept at MU. At MU, mindfulness has been long established for student and staff wellbeing as it was introduced in the early 2000s by Assoc. Prof. Craig Hassed. Since then mindfulness has been part of the core curriculum in medical programmes at the university for over 20years and has also been incorporated into other disciplines, such as psychology. In comparison, KCL was in the early stages of developing and implementing their Student Mental Health and Wellbeing Strategic Plan at this time of this research. This relatively recent prioritisation of student wellbeing and access to wellbeing initiatives at KCL highlights the large contextual differences of student wellbeing at the two universities. Coxon et al. (under review) provide a detailed commentary on the contextual differences between KCL and MU, and describes the action research undertaken at KCL as part of this study.

Limitations to the design of the intervention include firstly, a lack of introduction to mindfulness provided to participants. This was a decision made due to time constraints of the brief intervention. Secondly, a follow-up of possible adverse effects was not provided to participants. There is some research reporting negative effects of mindfulness, such as for those having a traumatic background; however, there has been little empirical evidence on the prevalence and severity of significant adverse events (Creswell, 2017). An important advantage of mindfulness is that for the general population, potential adverse events are rare and mild and may include unpleasant reactions, such as agitation and discomfort (Creswell, 2017). In a recent systematic review adverse events, most commonly anxiety was experienced in approximately 8% of participants, which is similarly to prevalence experienced in psychotherapy in general (Farias et al., 2020). Nevertheless, it remains a limitation of the study design that there was a lack of support structure in place to provide support to participants if they did experience negative effects of the mindfulness intervention.

Limitations to the research design include: (1) lack of data (beyond self-report) measuring intervention engagement, (2) procedural issue in data collection of the WEMWBS scale, (3) under-representative study discipline and gender distribution in the sample and (4) lack of data measuring negative intervention effects. Due to the low validity of a self-report item measuring intervention engagement, we were also unable to reliably measure the extent to which benefits are impacted by the amount of mindfulness practice. The procedural issue regarding the WEMWBS scale resulted in a comparison of 12 (MU) versus 14 (KCL) items. This limitation needs to be taken into consideration when interpreting and generalising the findings regarding the wellbeing measure. Thirdly, as we were only able to invite students who were undertaking a subject that was offered both on-campus and online, and at both universities, this sample recruitment strategy resulted in an uneven sample distribution. Specifically, the sample was heavily made up of psychology students, and as a result, more females participated than males. These two factors may have impacted our findings as psychology students from a helping profession may be more motivated to focus on and address their mental wellbeing. Although the sample was not stratified when allocating study conditions, the male participants in our sample were roughly distributed across the control and intervention groups. Finally, collecting data on any negative participant experiences would provide a more balanced discussion.

Limitations to the intervention protocol include as: (1) gender and delivery differences in the presentation of the mindfulness led exercises at MU and KCL and (2) differences in intervention duration between on-campus and online students. The participants’ experience of mindfulness may have been impacted by the exercises being led by a male (MU) compared to female (KCL), as well as possible differences in the delivery of the exercises. While we provide a rationale for why on-campus students undertook a 12-week intervention and the online students a 6-week intervention due to each matching the length of their study period (see section ‘Design’), we do acknowledge that such differences in duration may have confounded the findings.

#### Future Research

In future studies, we suggest targeting students from non-helping professions, that are male, exploring the sub-population of international students, and recruiting a more balanced sample with both younger online, and older on-campus students. Finally, due to the correlations between change in outcome measures, baseline levels and the effect of the intervention, future studies should aim to take all of these variables into account. Disentangling and understanding the subtle but the true complex relationships in the model require further experiments and larger sample sizes.

### A Comment on the Educational Implications and Future Directions

The MBI piloted in this study was fully online (accessed *via* students’ university LMS), asynchronous and consisted of short, audio-guided exercises. Although this brief pilot intervention significantly impacted on psychological outcomes, gains could potentially be greater if a more intensive programme is utilised. Yet we also acknowledge this brief intervention was effective and more intensive programmes may introduce other challenges, such as an even greater level of drop-out and attrition. Finding the right balance between evidence-based intervention design and maintaining participant engagement and retention requires further investigation.

We also take this opportunity to raise two wider comments and implications of student wellbeing initiatives. Firstly, gaining initial interest and engagement from students for wellbeing-related initiatives can be challenging. Students who have pre-existing interests in health and wellbeing, or those studying related disciplines may be more inclined to participate in self-management interventions, such as the case in the current study. Students who have little prior interest on this topic may be significantly less likely to pay attention to or consider taking part in wellbeing initiatives. We know from existing research that all students may be susceptible to lower mental health given the stressful nature of university life and that any group or type of students may be affected. We contend that it is important that we place attention on and investigate how to engage *all* students, and not just those that may be inherently ‘easier’ to engage.

Furthermore, maintaining engagement in a self-management initiative and reducing drop-out is a challenge we continue to face at universities. At times of high stress in the semester (e.g. assignments and end of term exams), students may de-prioritise actively addressing their wellbeing. For example, sleep and healthy eating habits may be reduced, and so too may participating in self-managed wellbeing activities. Again, this real-life challenge for university students needs to continue to be discussed and addressed.

#### Future Directions

As part of a larger study, free-text feedback was gathered from the sample in this current study. This qualitative data primarily suggested the inclusion of more variety and longer audio exercises, and the option of listening to a male and female presenter. Reasons why students stopped engaging in the activities revealed a common trend of students expressing ‘not having enough time’ or ‘getting too busy’. This supports our notion in the paragraph above, regarding maintaining engagement throughout the semester. Further details on qualitative feedback is available in Coxon et al. (under review). After revising the intervention based on the aforementioned feedback, academics at MU are currently trialling the programme during COVID-19 as part of an online orientation resource that is embedded material in students’ learning architecture, equally targeting students from all disciplines and accessible to students year-round.

## Conclusion

In summary, our study demonstrated a brief, online, asynchronous and guided MBI significantly improved university students’ levels of wellbeing, stress and mindfulness over the course of one semester. The intervention condition predicted up to 12% of variability in change in psychological outcomes, after controlling for baseline levels, age and study mode, with small–medium effect sizes. This research provides strong evidence for the use of MBIs as appropriate preventative approaches to supporting student mental health and for being effective in supporting special sub-groups of students, such as mature and returning students. Finally, practical implications of this study include its support for the use of digital wellbeing interventions for aiding study-related stresses and encouraging students to actively manage their psychological wellbeing in both on-campus and online modes.

## Data Availability Statement

The datasets generated for this study are available on request to the corresponding author.

## Ethics Statement

The studies involving human participants were reviewed and approved by the Monash University Human Research Ethics Committee (Monash University) and the King’s College London Psychiatry, Nursing and Midwifery Research Ethics Subcommittee (King’s College London). The participants provided their written informed consent to participate in this study.

## Author Contributions

JC, KD, AC, and SM conceptualised and designed the study protocol. JC and AC collected the data. JC and IH conducted data analysis and interpretation. MM and SM provided expert guidance and data interpretation. JC prepared the first draft of the manuscript. All authors contributed to the revision of the manuscript and approved the submitted version.

## Funding

This study was funded by the School of Psychological Sciences, Monash University to support the research collaboration between Monash University and King’s College London.

## Conflict of Interest

The authors declare that the research was conducted in the absence of any commercial or financial relationships that could be construed as a potential conflict of interest.

## Publisher’s Note

All claims expressed in this article are solely those of the authors and do not necessarily represent those of their affiliated organizations, or those of the publisher, the editors and the reviewers. Any product that may be evaluated in this article, or claim that may be made by its manufacturer, is not guaranteed or endorsed by the publisher.

## Footnotes

^1^The drop-out rate of KCL participants is presented in full in Coxon et al. (under review).
