# Supplementary material for: An Evaluation of an Online Brief Mindfulness-Based Intervention in Higher Education: A Pilot Conducted at an Australian University and a British University
Source: Front Psychol. 2021 Oct 28;12:752060. doi: 10.3389/fpsyg.2021.752060 (PMC8582350; doi:10.3389/fpsyg.2021.752060)
Supplement: Supplementary file 1 [file Table_1.DOCX]

Supplementary Material 1

*Participant Drop-Out Analyses on Outcome Measures at Baseline*

|  | Dropped out | |  | Did not drop out | |  |  |  |  |
| --- | --- | --- | --- | --- | --- | --- | --- | --- | --- |
|  | *n* | *M (SD)* |  | *n* | *M (SD)* | *t* | *df* | *p* | Hedge’s *g*[95% CI] |
| WEMWBS | 273 | 3.30 (0.57) |  | 151 | 3.36 (0.58) | -1.03 | 422 | 0.303 | 0.10 [-0.17,0.05] |
| PSS | 268 | 20.33 (6.42) |  | 148 | 19.82 (6.85) | 0.76 | 414 | 0.449 | 0.08 [-0.81, 1.83] |
| MAAS | 271 | 3.72 (0.73) |  | 149 | 3.61 (0.80) | 1.43 | 418 | 0.154 | 0.15 [-0.04, 0.26] |

*Note.* Dropped out = participants that did not complete the Time 2 questionnaire. Did not drop out = participants that completed Time 1 and Time 2 questionnaires. WEMWBS = Warwick Edinburgh Wellbeing Scale. PSS = Perceived Stress Scale. MAAS = Mindful Attention Awareness Scale. CI = confidence interval.

Supplementary Material 2

*Group Differences by Age and Study Mode on Outcome Measures at Baseline*

|  | Age | | |  |  |  |  |
| --- | --- | --- | --- | --- | --- | --- | --- |
|  | 18 – 35  *n* = 101 |  | 36+  *n* = 46 |  |  |  |  |
|  | *M (SD)* |  | *M (SD)* | *t* | *df* | *p* | Hedge’s *g*[95% CI] |
| WEMWBS | 3.29 *(0.59)* |  | 3.49 *(0.53)* | -1.97 | 145 | .051 | 0.35 [-0.40, 0.0006] |
| PSS | 20.96 *(6.82)* |  | 17.26 *(6.33)* | 3.12 | 145 | .002 | 0.55 [1.35, 6.05] |
| MAAS | 3.50 *(0.71)* |  | 3.86 *(0.95)* | -2.27 | 68.47 | .026 | 0.45 [-0.67, -0.04] |
|  | Study Mode | | |  |  |  |  |
|  | Online  *n* = 75 |  | On-campus  *n* = 72 |  |  |  |  |
|  | *M (SD)* |  | *M (SD)* | *t* | *df* | *p* | Hedge’s *g*[95% CI] |
| WEMWBS | 3.42 *(0.52)* |  | 3.28 *(0.63)* | 1.44 | 137.39 | .153 | 0.24 [-.051, 0.33] |
| PSS | 18.49 *(6.57)* |  | 21.17 *(6.95)* | -2.40 | 145 | .018 | 0.40 [-4.88, -0.47] |
| MAAS | 3.65 *(0.91)* |  | 3.57 *(0.69)* | .64 | 137.52 | .525 | 0.10 [-0.18, 0.35] |

WEMWBS = Warwick Edinburgh Wellbeing Scale. PSS = Perceived Stress Scale. MAAS = Mindful Attention Awareness Scale. CI = confidence interval.

|  | Change in WEMWBS | | | |  | Change in PSS | | | |  | Change in MAAS | | | |
| --- | --- | --- | --- | --- | --- | --- | --- | --- | --- | --- | --- | --- | --- | --- |
|  | *n* | *M* (SD) | *p* | Hedge’s *g* [95% CI] |  | *n* | *M* (SD) | *p* | Hedge’s *g* [95% CI] |  | *n* | *M* (SD) | *p* | Hedge’s *g* [95% CI] |
| Condition |  |  |  |  |  |  |  |  |  |  |  |  |  |  |
| Control | 69 | -0.43 (0.67) | **<.001***** | 0.80^c^ [-0.64, -0.27] |  | 68 | 2.66 (6.81) | **<.001***** | 0.61^b^ [1.71, 5.69] |  | 68 | -0.24 (0.74) | **<.001***** | 0.83^c^ [-0.85, -0.38] |
| Intervention | 82 | 0.03 (0.48) |  |  |  | 80 | -1.04 (5.44) |  |  |  | 81 | 0.37 (0.73) |  |  |
| Institution |  |  |  |  |  |  |  |  |  |  |  |  |  |  |
| KCL | 21 | -0.14 (0.48) | 0.72 | 0.10 [-0.24, 0.34] |  | 21 | 0.81 (4.70) | 0.91 | 0.03 [-2.80, 3.14] |  | 22 | 0.13 (0.57) | 0.83 | 0.05 [-0.32, 0.40] |
| MUA | 130 | -0.19 (0.64) |  |  |  | 127 | 0.64 (6.61) |  |  |  | 127 | 0.09 (0.82) |  |  |
| Age |  |  |  |  |  |  |  |  |  |  |  |  |  |  |
| 18 - 35 | 108 | -0.26 (0.62) | **.020*** | 0.41^a^ [-0.45,-0.04] |  | 101 | 1.61 (6.28) | **.007**** | 0.48^a^ [0.83, 5.17] |  | 102 | 0.04 (0.77) | **.003**** | 0.43^a^ [-0.68, -0.14] |
| 36+ | 48 | -0.009 (0.59) |  |  |  | 47 | -1.38 (6.11) |  |  |  | 47 | 0.37 (0.76) |  |  |
| Gender |  |  |  |  |  |  |  |  |  |  |  |  |  |  |
| Male | 15 | -0.08 (0.44) | 0.45 | 0.20 [-0.20,0.46] |  | 14 | 1.00 (5.94) | 0.84 | 0.06 [-3.20, 3.94] |  | 14 | 0.28 (0.50) | 0.34 | 0.26 [-0.24, 0.65] |
| Female | 134 | -0.20 (0.63) |  |  |  | 132 | 0.63 (6.47) |  |  |  | 133 | 0.07 (0.82) |  |  |
| Study mode |  |  |  |  |  |  |  |  |  |  |  |  |  |  |
| Online | 78 | 0.002 (0.55) | **<.001***** | 0.66^b^ [0.19, 0.57] |  | 76 | -1.27 (5.60) | **<.001***** | 0.65^b^ [-5.93, -1.99] |  | 76 | 0.29 (0.74) | **.001***** | 0.52^b^ [0.17, 0.66] |
| On-campus | 73 | -0.39 (0.63) |  |  |  | 72 | 2.69 (6.51) |  |  |  | 73 | -0.12 (0.79) |  |  |
| Level |  |  |  |  |  |  |  |  |  |  |  |  |  |  |
| Undergrad | 127 | -0.20 (0.64) | 0.37 | 0.19 [-0.39, 0.15] |  | 124 | 0.80 (6.60) | 0.56 | 0.13 [-1.97, 3.64] |  | 124 | 0.08 (0.82) | 0.63 | 0.10 [-0.43, 0.26] |
| Postgrad | 24 | -0.08 (0.50) |  |  |  | 24 | -0.04 (4.96) |  |  |  | 25 | 0.16 (0.64) |  |  |
| Meditation |  |  |  |  |  |  |  |  |  |  |  |  |  |  |
| No | 38 | -0.11 (0.54) | 0.46 | 0.15 [-0.15, 0.32] |  | 36 | 0.22 (5.73) | 0.66 | 0.09 [-3.10, 1.98] |  | 36 | 0.25 (0.81) | 0.25 | 0.22 [-0.13, 0.50] |
| Yes | 92 | -0.20 (0.64) |  |  |  | 92 | 0.78 (6.81) |  |  |  | 93 | 0.07 (0.82) |  |  |
| Mindfulness |  |  |  |  |  |  |  |  |  |  |  |  |  |  |
| No | 26 | -0.15 (0.70) | 0.79 | 0.06 [-0.24,0.32] |  | 26 | 0.58 (7.38) | 0.97 | 0.006 [-3.09, 3.00] |  | 26 | 0.18 (0.78) | 0.67 | 0.09 [-0.30, 0.47] |
| Yes | 84 | -0.19 (0.61) |  |  |  | 82 | 0.62 (6.65) |  |  |  | 82 | 0.10 (0.89) |  |  |

Supplementary Material 3

*Group Differences for Change in Well-Being, Stress and mindfulness Between Time 1 and Time 2*

*Note.* KCL = King’s College London. MUA = Monash University Australia. WEMWBS = Warwick Edinburgh Wellbeing Scale. PSS = Perceived Stress Scale. MAAS = Mindful

Attention Awareness Scale. Change = Time 2 - Time 1. CI = confidence interval. Significant findings are in bold.

Hedge’s *g* effect size = ^a^small, ^b^medium, ^c^large.

* *p* < .05. ** *p* < .01. *** *p* < .001.

Supplementary Material 4

*P-values for Model Comparisons*

|  | Model 2 vs. Model 1 | Model 3 vs. Model 2 | Model 2a vs. Model 1a |
| --- | --- | --- | --- |
| Change in WEMWBS | .004** | .356 | .025* |
| Change in PSS | .043* | .032* | .017* |
| Change in MAAS | .034* | .125 | .000*** |

*Note.* WEMWBS = Warwick Edinburgh Wellbeing Scale. PSS = Perceived Stress Scale. MAAS = Mindful

Attention Awareness Scale.

This table depicts the *p*-values of regression model comparisons. Each *p*-value is based on an ANOVA F-test for the difference between two nested linear regression models. The null hypothesis in each test can be regarded as "the true coefficients for all the additional x-variables in the more complex model are zero". A low *p*-value (for example below 0.05) can be regarded as evidence against this hypothesis.

The dependent variable in all models for a particular row is indicated in the first column. Model 1 includes the following x-variables: a constant, the baseline value corresponding to the dependent variable, Condition, Age and Study mode. Model 2 is the same as Model 1, except that the baseline values of the two other outcome variables are added. Model 3 is the same as Model 2 except that an interaction term between each baseline value and Condition is added.

Model 1a includes all the x-variables that do not involve Condition (a constant; baseline values of WEMWBS, PSS and MAAS; Age; and Study mode). Model 2a is the same as Model 1a but includes Condition and the interactions between Condition and each of the baseline values.

The interaction terms are defined as the dummy variable for Condition (coded as 1 for the treatment group and 0 otherwise) multiplied by the value of the respective baseline measurement.

* *p* < .05. ** *p* < .01. *** *p* < .001.
